# Changes in Gene Expression Patterns of Circadian-Clock, Transient Receptor Potential Vanilloid-1 and Nerve Growth Factor in Inflamed Human Esophagus

**DOI:** 10.1038/srep13602

**Published:** 2015-09-04

**Authors:** Shu-Chuan Yang, Chien-Lin Chen, Chih-Hsun Yi, Tso-Tsai Liu, Kun-Ruey Shieh

**Affiliations:** 1 General Education Center, Tzu Chi College of Technology, Hualien, Taiwan; 2 Department of Medicine, Buddhist Tzu Chi General Hospital, Hualien, Taiwan; 3 School of Medicine, Tzu Chi University, Hualien, Taiwan; 4 Department of Physiology, Tzu Chi University, Hualien, Taiwan; 5 Institute of Physiological and Anatomical Medicine, Tzu Chi University, Hualien, Taiwan

## Abstract

Circadian rhythm is driven by the molecular circadian-clock system and regulates many physiological functions. Diurnal rhythms in the gastrointestinal tract are known to be related to feeding pattern, but whether these rhythms are also related to the gastrointestinal damage or injuries; for example, gastroesophageal reflux disease (GERD), is unclear. This study was conducted to determine whether expression of circadian-clock genes or factors involved in vagal stimulation or sensitization were altered in the esophagus of GERD patients. Diurnal patterns of *PER1*, *PER2*, *BMAL1*, *CRY2*, *TRPV1*, and *NGF* mRNA expression were found in patient controls, and these patterns were altered and significantly correlated to the GERD severity in GERD patients. Although levels of *CRY1*, *TIM*, *CB1*, *NHE3*, *GDNF*, and *TAC1* mRNA expression did not show diurnal patterns, they were elevated and also correlated with GERD severity in GERD patients. Finally, strong correlations among *PER1*, *TRPV1*, *NGF* and *CRY2* mRNA expression, and among *PER2*, *TRPV1* and *CRY2* expression were found. Expression levels of *CRY1* mRNA highly correlated with levels of *TIM*, *CB1*, *NHE3*, *GDNF* and *TAC1*. This study suggests that the circadian rhythm in the esophagus may be important for the mediation of and/or the response to erosive damage in GERD patients.

Gastroesophageal reflux disease (GERD) is a common disorder that occurs in 5–20% of the population in the world, and its prevalence appears to be increasing^1^. Patients with GERD usually have symptoms, such as heartburn, acid regurgitation and/or esophageal mucosal damage, that may induce long-term and severe complications, such as Barrett’s esophagus^2^,^3^. Although Barrett’s esophagus is associated with severe GERD and an increased risk of esophageal adenocarcinoma, most patients with Barrett’s esophagus do not develop esophageal cancer^2^.

Excessive gastric juice cause reflux esophagitis through the incompetence of the lower esophageal sphincter. Acid refluxate stimulates the sensory afferents and is an important element underlying symptom generation in GERD patients. The proliferation index (Ki-67)^4^,^5^ and a cell cycle-related protein (p53)^6^ have been shown to be positively correlated with GERD severity in GERD patients. Acid stimulation in GERD symptoms has been determined to act through the activation of proton-gated and acid-sensing ion channels, such as the transient receptor potential vanilloid receptor 1 (TRPV1)^7^,^8^. Elevations in TRPV1 expression have been found in the esophageal mucosa of GERD subjects^9^,^10^,^11^ and in rats with reflux-induced esophagitis^12^. Both nerve growth factor (NGF) and glial cell derived neurotrophic factor (GDNF)^13^,^14^ play important roles in the regulation of vagal unmyelinated sensory C-fibers, which also express the TRPV1 receptor to sense the chemical and/or thermal stimuli^15^. An increase in TRPV1 expression has also been shown to be related to the GDNF and NGF elevations in patients suffering from fecal urgency^16^. Furthermore, several factors, such as cannabinoid receptors (CB)^17^,^18^, Na/H exchangers (NHE)^19^ and substance P^12^,^20^, which is encoded by *protachykinin-1* (*TAC1*), have been found to be related to GERD symptoms in animal models or patients. Therefore, whether the levels of *CB1*, *CB2*, *NHE1*, *NHE3*, *TRPV1*, *NGF*, *GDNF* and *TAC1* gene expressions in GERD patients were altered and correlated to the severity of GERD symptoms was examined in this study.

Circadian rhythmicity exists in many behavioral and physiological activities in mammals, and allows the organism to predict and respond to anticipated environmental demands and serves a vital role by matching physiological functions^21^,^22^. The mammalian circadian rhythm is driven by the molecular circadian-clock system, including the *Per1* (period 1), *Per2*, *Per3*, *Cry1* (cryptochrome 1), *Cry2*, *Bmal1* (brain and muscle aryl-hydrocarbon receptor nuclear translocator-like protein-1), *Clock* (circadian locomotor output cycles kaput) and *Timeless* (*Tim*)^21^,^22^. This molecular circadian-clock system exists throughout the whole body, including the central circadian pacemaker, the suprachiasmatic nucleus, and many peripheral tissues, such as the heart, kidney, liver and gastrointestinal tract^23^,^24^,^25^,^26^. Although the physiological role of the circadian-clock genes in all tissues is not fully understood, their disruption affects on the progression of cancers, including breast, gastric and colorectal cancers^27^,^28^,^29^, and they have been postulated to have an effect on the regulation of gastrointestinal function^30^,^31^,^32^,^33^. Gating of the cell cycle by the circadian-clock system has also been reported^34^. Notably, one of the main complaints in GERD patients is a distinct burning sensation at night, which is attributed to nocturnal reflux^35^,^36^, and this finding reveals that diurnal pattern exist in GERD symptoms and are correlated with symptom severity^36^. GERD is the disease that is associated with acid damage and inflammation, and a recent study showed that circadian-clock system is susceptible to oxidative stress and inflammation^37^. Therefore, this study aimed to determine whether the gene expression levels of circadian-clock genes and several factors involved in vagal stimulation or sensitization were altered by the severity in inflamed human esophagus.

## Materials and Methods

### Subjects

The patient control group comprised asymptomatic subjects, who had a routine health checkup and did not have any esophageal reflux symptoms. GERD patients with symptoms of acid regurgitation and/or heartburn at least six months in duration were enrolled. Erosive esophagitis was classified according to the Los Angeles (LA) classification system^38^. Before or during the study, no subject took any treatments, including a proton pump inhibitor. Subjects who had diabetes, Barrett’s esophagus, esophageal strictures, eating disorders or sleep disorders; took antiepileptics or anticoagulants; or were pregnancy or nursing were excluded from the study. All experimental protocols in this study were carried out in accordance with approved guidelines by the Ethics Committee of Buddhist Tzu Chi General Hospital (Hualien, Taiwan). The written consent was received from each subject.

### Endoscopy

Two biopsy samples from the distal esophagus of each subject were taken during the endoscopic examination. One sample was for histological examination to verify the esophagitis conditions, and the other was for gene detection. Because the regular periods of endoscopic examination were 9–12 AM and 2–5 PM, the time points of biopsies from the subjects in this study were obtained within a 30-min window at 9 AM and 4 PM and referred to as the AM and PM groups, respectively. Each subject underwent a single endoscopic examination on the examination day. To achieve sampling constancy, the specimens were taken from a fixed position, which was approximately one inch above the squamocolumnar junction, using the standard biopsy forceps in all subjects during the endoscopic examination. The biopsies from GERD patients were obtained from positions between esophagitis erosions. The severity of the mucosal damage in GERD patients was evaluated according to the LA grading classification system^38^, and erosive damage in GERD patients was endoscopically defined by the erosions and/or ulcers in mucosal breaks.

### RNA isolation and real-time qPCR

After endoscopy, fresh specimens from biopsy were immediately stocked at 4 °C in RNA*later*^®^ solution (Ambion, Austin, TX, USA) as in previous studies^10^,^39^,^40^. The MasterPure^TM^ RNA Purification Kit (Epicentre, Madison, WI, USA), containing the RNase-Free DNase I, Proteinase K and RNase Inhibitor, was used for RNA isolation. The RNA was resuspended in DEPC-H_2_O, and for all samples, the 260/280 ratios were measured using spectrophotometry to assess RNA purity. Only samples with 260/280 OD ratios of 1.8–2.0 were used to examine the RNA integrity. RNA integrity was examined using an Agilent 2100 Bioanalyzer (Agilent Technologies, Santa Clara, CA, USA), and samples with an RNA integrity number over 7.0 and rRNA 28S/18S over 1.8 were used for reverse transcription.

Immediately after RNA extraction, the ImProm-II^TM^ Reverse Transcription System (Promega, Madison, WI, USA) as well as oligo(dT)_20_ and random hexamer primers were used for reverse transcription of all RNA samples into cDNA. Real-time qPCR was performed in triplicate with a Chromo4 Continuous Fluorescence Detector (Bio-Rad, Hercules, CA, USA) with 2 × Maxima^TM^ SYBR Green/ROX qPCR Master Mix (Thermo Scientific, Waltham, MA, USA) and 0.2 μM primers (listed in Table 1). All preparations were according to the manufacturer’s instructions and protocols. The primers were designed using Beacon Designer Software V7.0 (Bio-Rad) and based on the sequence information from the National Center for Biotechnology Information database. Relative expression levels were determined using the comparative quantification cycle (Cq) method, and normalized to reference genes, β-actin and GAPDH. Because the relative ratios of target genes with β-actin and GAPDH were similar, the present study showed only the data normalized to β-actin, as in our previous studies^10^,^39^,^40^. The relative change in genes expression was determined using the ΔΔC_T_ method and fold-change analysis.

### Statistical analysis

A post hoc test (Student-Newman-Keuls) subsequent to two-way analysis of variance (ANOVA) was used to determine the differences in mRNA expression among different groups of subjects. A Pearson’s correlation coefficient was applied to test correlations between levels of mRNA expression. A *P*-value of less than 0.05 was considered to be statistically significant.

## Results

In the study 66 subjects were enrolled between February and December 2012. The characteristics of these subjects are shown in Table 2. There were no significant age or sex difference between the patient control and GERD patient groups (Table 2; *P* > 0.05).

**Figure 1:**
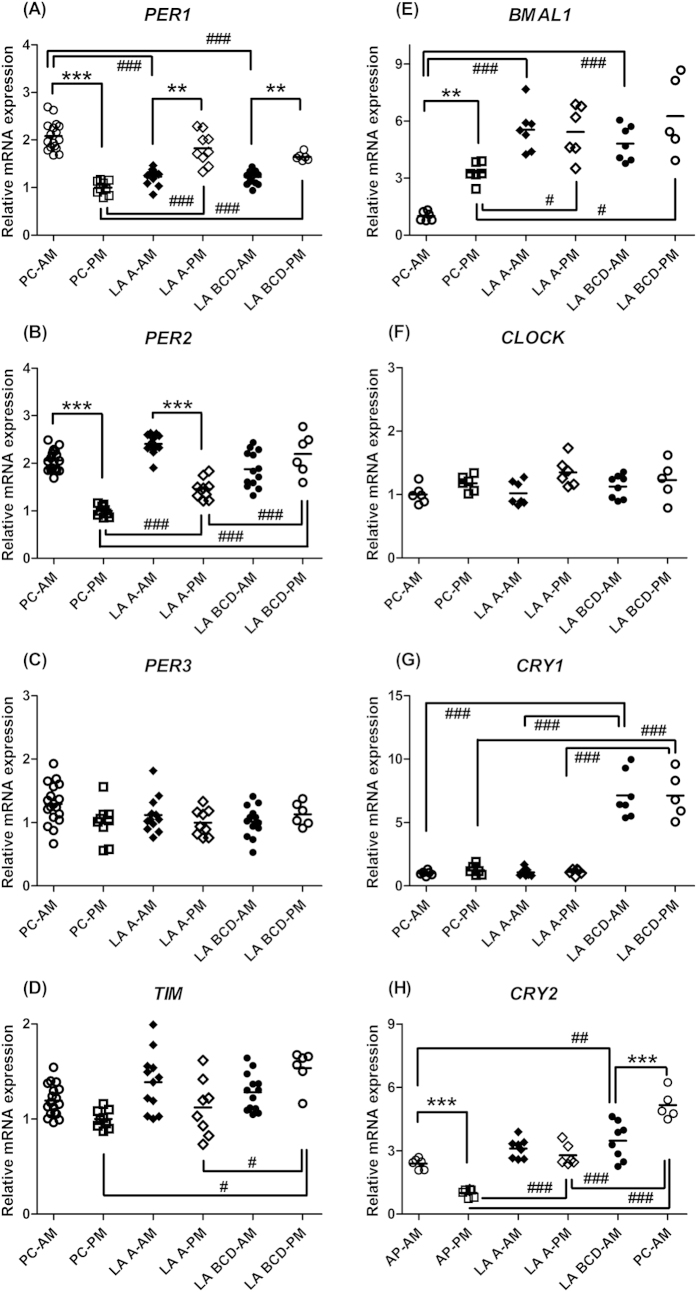
Analytical data of the expression of circadian-clock genes, (A) *PER1* (period1), (B) *PER2*, (C) *PER3*, (D) *TIM* (timeless), (E) *BMAL1* (brain and muscle aryl-hydrocarbon receptor nuclear translocator-like protein-1), (F) *CLOCK* (circadian locomotor output cycles kaput), (G) *CRY1* (cryptochrome 1), and (H) *CRY2* in the esophagus of patient controls (PC) and the patients with different Los Angeles (LA) classification grades (A–D) of gastroesophageal reflux disease. The biopsies from all subjects were divided between 9 AM and 4 PM time points and referred to as the AM and PM groups. **p < 0.01; ***p < 0.001 compared with the same groups in the other biopsy time point; ^#^*p* < 0.05; ^##^*p* < 0.01; ^###^*p* < 0.001 compared with the other groups in the same biopsy time point. Line represents the mean value.

Expression of *PER1* mRNA in the esophagus of the patient controls showed a diurnal pattern, which was higher in the morning and lower in the afternoon (Fig. 1A, *P* < 0.001). This diurnal expression pattern of *PER1* mRNA was affected by the severity of GERD conditions. In the subjects with either Grade A or Grades B-D GERD, the *PER1* mRNA expression in the esophagus still showed a diurnal pattern, but its pattern was inverted, i.e., lower level in the morning and higher level in the afternoon (Fig. 1A, *P* < 0.01). *PER2* mRNA expression in the esophagus of the patient controls also had a diurnal pattern that was higher in the morning and lower in the afternoon (Fig. 1B, *P* < 0.001). Expression pattern of *PER2* mRNA in the subjects with Grade A GERD was similar to that in the patient controls (Fig. 1B, *P* < 0.001); however, expression of *PER2* mRNA in the subjects with Grades B-D GERD was severely affected and not detected the rhythmic pattern (Fig. 1B, *P* > 0.05). At the same time, expression levels of *PER2* mRNA in the afternoon gradually increased with the severity of GERD classification (Fig. 1B, *P* < 0.001). A diurnal pattern of *PER3* gene expression in the esophagus was not detected, and the severity of GERD also did not affect the levels of expression (Fig. 1C, *P* > 0.05).

Although expression of *TIM* mRNA in the esophagus did not show a diurnal pattern, the expression levels in the afternoon were significantly increased by the severity of GERD (Fig. 1D, *P* < 0.05). *TIM* mRNA expression levels in the afternoon in the subjects with Grades B-D GERD were higher than the levels in the patient controls and in the subjects with Grade A GERD (Fig. 1D, *P* < 0.05). Expression of *BMAL1* mRNA in the esophagus showed a diurnal pattern, which was lower in the morning and higher in the afternoon in the patient controls (Fig. 1E, *P* < 0.01). This diurnal expression pattern of *BMAL1* mRNA was affected by the severity of GERD. In the subjects with Grade A or Grades B-D GERD, a diurnal pattern of *BMAL1* mRNA expression was undetectable and the levels in both the morning and the afternoon were elevated compared to those in the patient controls (Fig. 1E, *P* < 0.001 and *P* < 0.05, respectively). A diurnal pattern of *CLOCK* gene expression in the esophagus was undetectable, and the GERD conditions also did not affect the levels of expression (Fig. 1F, *P* > 0.05). A diurnal pattern of *CRY1* gene expression in the esophagus was also undetectable (Fig. 1G, *P* > 0.05), but the levels were significantly elevated in the subjects with Grades B-D GERD (Fig. 1G, *P* < 0.001).

Expression of *CRY2* mRNA in the esophagus of the patient controls showed a diurnal pattern, which was higher in the morning and lower in the afternoon (Fig. 1H, *P* < 0.001). This diurnal expression pattern of *CRY2* mRNA was affected by the severity of GERD. In the subjects with Grade A GERD, a diurnal pattern of *CRY2* mRNA expression was undetectable (Fig. 1H, *P* > 0.05) and the levels in the afternoon were significantly elevated compared to those in the patient controls (Fig. 1H, *P* < 0.001). In the subjects with Grades B-D GERD, a diurnal pattern of *CRY2* mRNA expression was detected, but its pattern was inverted, i.e., lower level in the morning and higher level in the afternoon (Fig. 1H, *P* < 0.01). Compared to the levels in the patient controls, the *CRY2* levels were significantly elevated in both the morning and the afternoon (Fig. 1H, *P* < 0.01 and *P* < 0.001, respectively).

**Figure 2:**
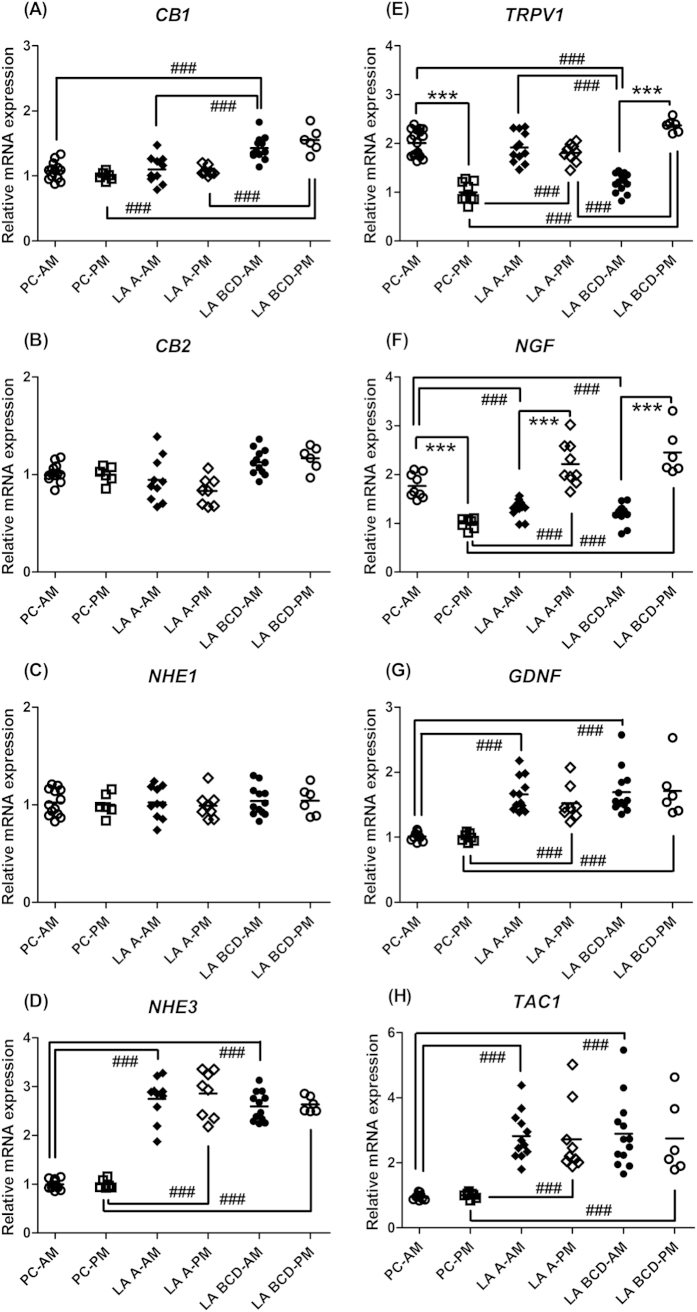
Analytical data of the expression of (A) *CB1* (cannabinoid receptor 1), (B) *CB2*, (C) *NHE1* (Na/H exchanger 1), (D) *NHE3*, (E) *TRPV1* (transient receptor potential vanilloid receptor 1), (F) *NGF* (nerve growth factor), (G) *GDNF* (glial derived neurotrophic factor), and (H) *TAC1* (protachykinin-1) genes in the esophagus of patient controls (PC) and the patients with different Los Angeles (LA) classification grades (A–D) of gastroesophageal reflux disease. The biopsies from all subjects were divided between 9 AM and 4 PM time points and referred to as the AM and PM groups. ***p < 0.001 compared with the same groups in the other biopsy time point; ^###^*p* < 0.001 compared with the other groups in the same biopsy time point. Line represents the mean value.

Diurnal variations in *CB1*, *CB2*, *NHE1*, *NHE3*, *GDNF* and *TAC1* mRNA expression in the esophagus were undetectable; however, the expression levels of these genes were affected by different grades of GERD (Fig. 2). Expression levels of *CB1* mRNA in the esophagus of the patient controls were similar to those in the subjects with Grade A GERD (Fig. 2A, *P* > 0.05), but the levels in the subjects with Grades B-D were elevated significantly in the both the morning and the afternoon (Fig. 2A, *P* < 0.001). Neither *CB2* nor *NHE1* mRNA expression in the esophagus was affected by GERD severity (Fig. 2B,C, *P* > 0.05). Expression levels of *NHE3* mRNA in the esophagus of patients with Grade A or Grades B-D GERD were higher than the levels of the control subjects (Fig. 2D, *P* < 0.001), but the levels were similar between the patients with different grades of GERD (Fig. 2D, *P* > 0.05). *TRPV1* mRNA expressions in the esophagus of the patient controls showed a diurnal pattern, with a higher level in the morning and a lower level in the afternoon (Fig. 2E, *P* < 0.001). The diurnal pattern of *TRPV1* mRNA disappeared in the patients with Grade A GERD (Fig. 2E, *P* > 0.05). A diurnal pattern of *TRPV1* mRNA reappeared in the patients with Grades B-D GERD (Fig. 2E, *P* < 0.001); however, this pattern was changed toward a lower level in the morning and a higher level in the afternoon (Fig. 2E, *P* < 0.001), so that the expression levels of *TRPV1* mRNA in the subjects with Grades B-D GERD in the morning were lower than those in the patient controls and those in the patients with Grade A GERD (Fig. 2E, *P* < 0.001). Expression levels of *TRPV1* mRNA in the afternoon were significantly and gradually elevated from Grade A to Grades B-D GERD (Fig. 2E, *P* < 0.001).

*NGF* mRNA expression in the esophagus of the patient controls also revealed a diurnal pattern, i.e., higher in the morning and lower in the afternoon (Fig. 2F, *P* < 0.001). Diurnal patterns of *NGF* mRNA expression in the patients with Grade A and those with Grades B-D GERD were also detectable, but they were reversed and were attenuated in the morning and elevated in the afternoon compared to the levels in the patient controls (Fig. 2F, *P* < 0.001). Therefore, the expression levels of *NGF* mRNA exhibited a difference between the patient controls and the GERD patients in either the morning or the afternoon (Fig. 2F, *P* < 0.001). There was no difference in the expression levels of *NGF* mRNA between the patients with Grade A and those with Grades B-D GERD in the either the morning or the afternoon (Fig. 2F, *P* > 0.05). In contrast, a rhythmic pattern was not detected in *GDNF* or *TAC1* mRNA expression in the esophagus of either the patient controls or the GERD patients (Fig. 2G,H, *P* > 0.05). Levels of *GDNF* or *TAC1* mRNA expression were significantly elevated in the GERD subjects (Fig. 2G,H, *P* < 0.001); however, there was no difference between the patients with Grade A and those with Grades B-D GERD (Fig. 2G,H, *P* > 0.05).

When expression levels of each gene within the group of genes with a rhythmic pattern were compared, *PER1* mRNA expression was significantly correlated with *TRPV1* expression (Table 3 and Figure S1A; r = 0.5329, *P* < 0.001) and with *NGF* expression (Table 3 and Figure S1B; r = 0.6103, *P* < 0.001), but not with *CRY2* expression (Table 3 and Figure S1C; r = 0.1874, *P* > 0.05). *PER2* mRNA expression was also correlated well with *TRPV1* expression (Table 3 and Figure S1D; r = 0.4745, *P* < 0.001) and with *CRY2* expression (Table 3 and Figure S1E; r = 0.4691, *P* < 0.01). *NGF* mRNA expression was also significantly correlated with *CRY2* expression (Table 3 and Figure S1F; r = 0.5198, *P* < 0.001) and with *TRPV1* expression (Table 3 and Figure S1G; r = 0.5656, *P* < 0.001), and *TRPV1* mRNA expression was correlated with *CRY2* expression (Table 3 and Figure S1H; r = 0.5519, *P* < 0.001).

When expression levels of each gene in the group of genes without a rhythmic pattern were compared, *CRY1* mRNA expression was highly correlated with *TIM* expression (Table 4 and Figure S2A; r = 0.4584, *P* < 0.01), *CB1* expression (Table 4 and Figure S2B; r = 0.6779, *P* < 0.001), *GDNF* expression (Table 4 and Figure S2C; r = 0.3272, *P* < 0.05), *NHE3* expression (Table 4 and Figure S2D; r = 0.3337, *P* < 0.05), and *TAC1* expression (Table 4 and Figure S2E; r = 0.4115, *P* < 0.05). *TIM* mRNA expression was correlated well with *CB1* expression (Table 4 and Figure S2F; r = 0.5665, *P* < 0.001), *GDNF* expression (Table 4 and Figure S2G; r = 0.5208, *P* < 0.001), *NHE3* expression (Table 4 and Figure S2H; r = 0.4962, *P* < 0.01), and *TAC1* expression (Table 4 and Figure S2I; r = 0.3468, *P* < 0.05). *CB1* mRNA expression was correlated well with *GDNF* expression (Table 4 and Figure S2J; r = 0.3533, *P* < 0.05) and *NHE3* expression (Table 4 and Figure S2K; r = 0.4048, *P* < 0.001), but not with *TAC1* expression (Table 4 and Figure S2L; r = 0.2653, *P* > 0.05). Additionally, *GDNF* mRNA expression was significantly correlated with *NHE3* expression (Table 4 and Figure S2M; r = 0.7871, *P* < 0.001) and *TAC1* expression (Table 4 and Figure S2N; r = 0.4260, *P* < 0.01), and *NHE3* expression was also correlated well with *TAC1* expression (Table 4 and Figure S2O; r = 0.6165, *P* < 0.001).

## Discussion

**Figure 3:**
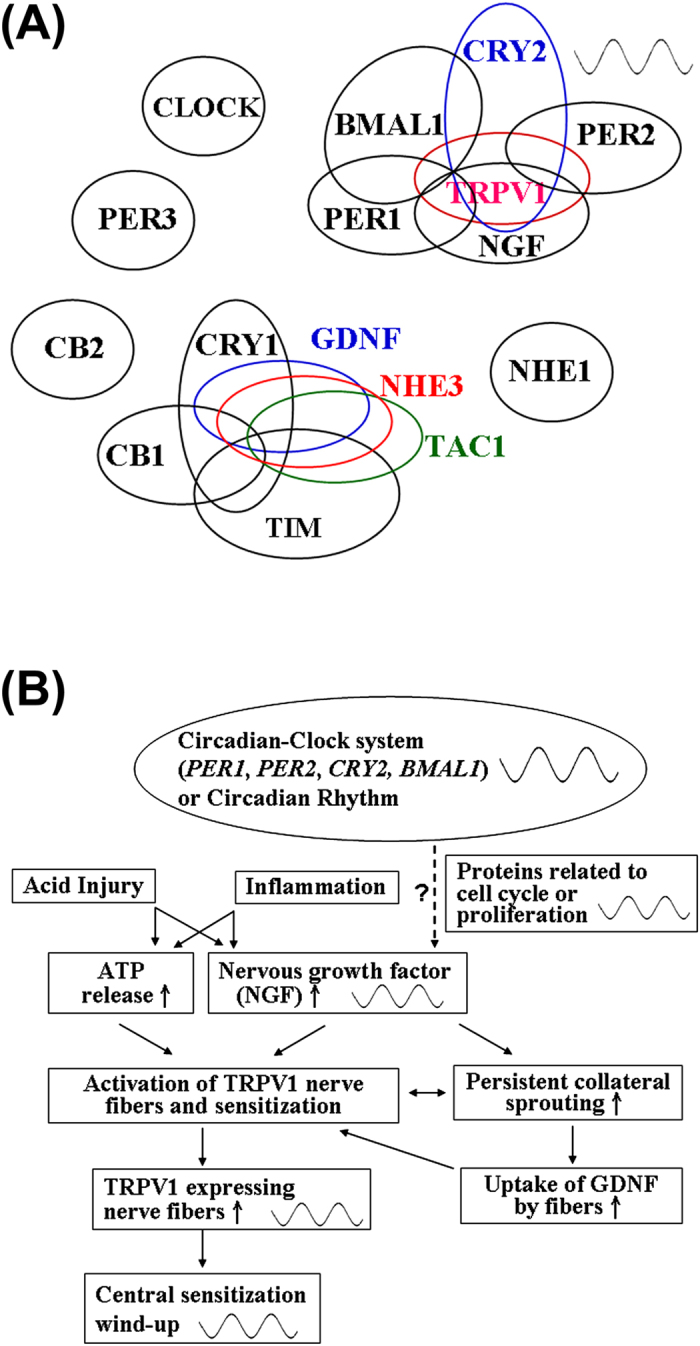
(**A**) Cluster correlations of total gene expression. Cluster correlations of gene expressions among *PER1* (period1), *PER2*, *PER3*, *CRY1* (cryptochrome 1), *CRY2*, *BMAL1* (brain and muscle aryl-hydrocarbon receptor nuclear translocator-like protein-1), *TRPV1* (transient receptor potential vanilloid receptor 1), *NGF* (nerve growth factor), *CB1* (cannabinoid receptor 1), *CB2*, *NHE1* (Na/H exchanger 1), *NHE3*, *GDNF* (glial derived neurotrophic factor), *TAC1* (protachykinin-1), *TIM* (timeless), and *CLOCK* (circadian locomotor output cycles kaput). (**B**) A schematic illustrating the proposed mechanism of esophageal sensitization and the relationship with the circadian-clock system or circadian rhythm in gastroesophageal reflux disease (GERD). Chronic acid injury and inflammation of the esophageal mucosa are initial damages that could induce a local increase in GDNF and NGF, which in turn might trigger pathways leading to persistent collateral sprouting and sensitization. Lowered thermal and/or mechanical thresholds and/or increased density of nerve fibers might be related to TRPV1 expression. The circadian-clock system, including PER1, PER2, CRY2 and BMAL1, may be involved in this elevation of TRPV1 expression through the regulation of protein expression in cell cycle or cell proliferation. Therefore, central sensitization and discomfort express diurnal patterns.

The circadian-clock systems play important roles in maintaining physiological homeostasis. Growing evidence shows that disruptions or impairment of the circadian-clock systems are related to diseases or syndromes, including diabetes and cancers. Although the changes of the circadian-clock system are found in breast, gastric and colorectal cancers^27^,^28^,^29^, few reports have investigated whether the circadian-clock system is related to the symptoms of gastrointestinal disorders, especially GERD. Furthermore, overexpression of a cell cycle-related protein (p53) has been reported in GERD patients^6^, and the circadian-clock system is involved in the gating of the cell cycle^34^. Therefore, this study is the first report to determine whether the expression of circadian-clock genes, and factors involved in vagal stimulation or sensitization in the esophagus was changed and correlated with the severity of the condition in GERD patients. Using the cluster correlations from the expression levels of these genes, these gene expression patterns were divided into three related groups, i.e., correlated with the severity in GERD and exhibited rhythmic patterns (*PER1*, *PER2*, *CRY2*, *BMAL1*, *TRPV1* and *NGF* mRNAs), correlated with the GERD severity but revealed no rhythmic pattern (*CRY1*, *TIM*, *CB1*, *GDNF*, *NHE3* and *TAC1* mRNAs), and unrelated to the severity of GERD (*CLOCK*, *PER3*, *CB2* and *NHE1* mRNAs) (Fig. 3A). Several major findings in this study are that (1) diurnal rhythmic patterns in the expression of the circadian-clock (*PER1*, *PER2*, *BMAL1* and *CRY2* mRNAs), *TRPV1* and *NGF* genes and their correlation with the severity of GERD classified by LA Grades were found; (2) high correlations among *PER1*, *TRPV1*, *NGF* and *CRY2* mRNA expression, and good correlations among *PER2*, *TRPV1* and *CRY2* expression were found; (3) increases of *CB1*, *NHE3*, *GDNF*, *TAC1*, *CRY1* and *TIM* mRNA expression levels related to the severity of GERD classified by LA Grades were found, but diurnal rhythmicity was not detected for these genes; and (4) high correlations among *CB1*, *NHE3*, *GDNF*, *TAC1*, *CRY1* and *TIM* mRNA expression were also found.

### Changes of diurnal patterns in *PER1*, *PER2*, *CRY2*, *BMAL1*, *TRPV1* and *NGF* mRNA expression in GERD patients

Rhythmic patterns of circadian-clock mRNA expression have been shown in human oral mucosa with higher expression of human *PER1* mRNA in the morning and lower expression in the afternoon^41^. Additionally, lower expression of *BMAL1* mRNA in the morning and higher expression of *BMAL1* mRNA in the afternoon were also shown in the same study^41^. Although the present study examined the circadian-clock gene expression in human esophageal mucosa, the expression patterns of *PER1* and *BMAL1* are similar to those in the previous study^41^. On the other hand, a recent study showed higher levels of *BMLA1* mRNA expression in the morning and higher levels of *PER2* mRNA expression in the afternoon in human oral mucosa^42^. Therefore, the data in this study were similar to those previously reported by Bjarnason *et al.*^41^ but not to those reported by Zieker *et al.*^42^. In this study, we did not detect a pattern in *CRY1* mRNA expression in human esophagus; however, a previous study showed similar patterns for *CRY1* and *BMAL1* mRNAs, which were the lower in the morning and higher in the afternoon, in human oral mucosa^41^. Possible explanations for the difference between our study and the previous study are that there are too few time points to detect the patterns in this study or that the *CRY1* mRNA has the different patterns in different tissues (oral vs esophageal mucosa). A previous study has shown that the rhythmic patterns of *Per1* and *Per2* mRNA expression disappeared in tumor cells of breast tumor bearing mice^43^. A connection between circadian disruption and cancer predisposition in *Per2* mutant mice has also been reported^44^. However, another study indicates that dual knockout of the circadian-clock genes *Cry1* and *Cry2* in mice does not necessarily predispose mice to cancer^45^. The present study did not focus on the predisposition to esophageal cancer with the disruption of circadian-clock gene expression in human subjects because, although Barrett’s esophagus is associated with severe GERD and with an increased risk of esophageal adenocarcinoma, most patients with Barrett’s esophagus do not develop esophageal cancer^2^. Only 5–10% of GERD patients develop Barrett’s esophagus, and 1% of patients with Barrett’s esophagus develop esophageal cancer^2^. This study showed that the rhythmic patterns and levels of *PER1*, *PER2*, *BMAL1* and *CRY2* mRNA expression in the esophagus were affected by the severity of GERD. Although the *PER1* mRNA expression in the esophagus of both the patient controls and the GERD patients show diurnal rhythms, the patterns are different and one is the inverse of the other. The *PER2* mRNA expression in the esophagus of the subjects with Grades B-D GERD was severely affected and even did not detect its rhythmic pattern. The afternoon levels of both *PER1* and *PER2* mRNAs were significantly elevated in the GERD patients. The rhythmic pattern of *BMAL1* mRNA in the esophagus disappeared in the subjects with Grades B-D GERD and the levels were significantly elevated. The rhythmic pattern of *CRY2* mRNA in the esophagus was affected by the severity of the condition in the GERD subjects and showed a loss of rhythmicity and even an inverted pattern. Furthermore, our preliminary data showed that the rhythmic patterns of *PER1* and *PER2* mRNAs were restored in two subjects with a history of GERD who recently completely recovery after medical treatments (data not shown). The sample size was too small; however, based on our data and those of previous studies, our conservative inference is that the circadian-clock system in the esophagus is responsive to the acid damage and even perceptive of vagal stimulation or sensitization.

Although Tim protein was initial identified in *Drosophila* to function in the regulation of circadian rhythmicity, the functions of mammalian Tim protein are cell cycle related^46^,^47^. Previous studies have shown that *TIM* and *CLOCK* mRNAs in the human oral mucosa did not reveal diurnal or circadian rhythmicity^41^,^42^, which is confirmed by our results in the esophageal mucosa. A previous study has also shown that down-regulation of human TIM protein compromises replication and intra-S checkpoints^47^. Expression of *TIM* mRNA did not appear rhythmic in the esophagus of the patient controls or the GERD patients, but elevation of *TIM* mRNA levels in the subjects with LA Grades B-D GERD in the afternoon were found in this study. Our finding implied that human *TIM* was not related to regulation of circadian rhythmicity but might be related to the cell cycle. PERs proteins activate the *p16-INK4A* cell cycle checkpoint gene in a circadian manner^48^. The DNA damage response may be very different between cells *in vitro* and in an intact organism *in vivo* in which various inputs are proposed to regulate homeostasis^49^. The data in this study indicate that esophageal tissues in GERD patients persist without normal patterns in circadian-clock gene expression, and this change in pattern might be related to the changes in the cell cycle, which raises the therapeutic implication that more complete circadian-clock control within cells might help to control diseases or eliminate symptoms. Whether the expression of circadian-clock and cell cycle-related mRNAs/proteins are linked to the inflamed esophagus in GERD patients still needs further study.

TRPV1 is a receptor in the acid-sensitive ion channel family, closely related to visceral sensitivity, and is expressed on both neural and epithelial cells. TRPV1 activation induces both inflammation and the sensation of burning pain by the release of substance P, which is encoded by *TAC1*^50^. An increase in TRPV1 expression has also been proposed to be related to the NGF and GDNF elevations in patients suffering from fecal urgency^16^. Several studies have also shown that *TRPV1*, *NGF*, *GDNF* and *TAC1* mRNAs or their proteins are related to gastrointestinal disorders including GERD^9^,^10^,^13^,^14^. However, there was no report as to whether expression of these gene showed diurnal patterns and was related to GERD conditions. In this study, the expression levels of *TRPV1* and *NGF* mRNAs exhibit diurnal patterns in the esophagus of the patient controls, and the patients with LA Grade A or Grades B-D GERD. Notably, the *TRPV1* and *NGF* mRNA expression in patients with LA Grades B-D GERD show an inverse diurnal pattern compared to the patient controls.

A recent double-blind, crossover study shows that the acid-suppresser, rabeprazole more effectively eased symptoms in patients with GERD in the evening^51^. Our present study provides the supportive and underlying information for this therapeutic effect and implies that the inverse diurnal patterns of *TRPV1* and/or *NGF* gene expression might contribute to the hypersensitivity in the evening or at night in patients with severe GERD. Interestingly, the proximal promoter of the rat *preprotachykinin-A* gene, which is similar to the human *TAC1* gene, contains an E-box site^52^. This proximal E-box modulates NGF effects on dorsal root ganglia^52^. The E-box is one of the transcription factor families associated with circadian rhythm in most tissues, and a BMAL1 and CLOCK protein complex binds to the E-box^21^,^22^. Furthermore, an upstream regulatory region of the TRPV1 gene that is regulated by NGF has been found and suggests that an increase of TRPV1 expression under conditions of tissue injury and/or inflammation is in part through a transcription-dependent mechanism and NGF-produced conditions^53^. Therefore, the expression of the circadian-clock (*PER1*, *PER2*, *BMAL1* and *CRY2*), *TRPV1* and *NGF* genes in the esophagus shows a diurnal rhythmic pattern and is correlated with the severity of GERD in patients (Table 3, Figure S1 and Fig. 3A).

### Increases of *CB1*, *NHE3*, *GDNF*, *TAC1*, *CRY1* and *TIM* mRNA expression levels in GERD patients

Rhythmic patterns of *CB1*, *NHE3*, *GDNF*, *TAC1*, *CRY1* and *TIM* mRNA expression in the human esophagus were not detected in this study. Although several factors involved in vagal stimulation or sensitization of GERD patients or animal models have been reported, there is little discussion of the relationships of the expression of these genes. Recent studies have shown that CB1 and CB2 receptors exist in the myenteric plexus of the lower esophageal sphincter in humans^17^, and a CB1 receptor agonist decreases relaxation of the transient lower esophageal sphincter in dogs and humans^18^,^54^. However, the higher or lower expression of *CB1* mRNA or its receptor in gastrointestinal diseases are controversial. CB1 receptors are upregulated during intestinal inflammation^55^, but CB1 mRNA expression is decreased in GERD patients^56^. The present data together with previous studies supported the therapeutic implication that levels of CB1 receptor are increased in the human, inflamed esophagus, and a CB1 receptor agonist might be beneficial for treatment.

One of the protective mechanisms evolved by cells against intracellular acidification is mediated by the NHE family to extrude protons from the cytoplasm, including the gastrointestinal tract^57^. Increased *NHE1* mRNA in patients with GERD has been reported^19^, and robust expression of NHE1 protein in Barrett’s esophagus but an absence of NHE1 in normal epithelium also has been shown^58^. In this study, we did not find that the levels of *NHE1* and *NHE3* mRNA in the esophagus reveal diurnal patterns and there was no change in levels of *NHE1* mRNA in patients with GERD. Levels of *NHE3* mRNA were elevated in the esophagus of both subjects with LA Grade A and Grades B-D GERD. Because only NHE3 has been found to traffic between the plasma membrane and the recycling endosomes and tends to acidify the early endosomes and perhaps secretory granules^59^, elevation of *NHE3* mRNA might contribute to increase transmitter(s) release, for example, GDNF, Substance P and/or ATP, in GERD patients. Although the levels of *GDNF* and *TAC1* mRNA expression do not show diurnal patterns, in patients with Grade A and those with Grades B-D GERD, the expression levels of these genes are higher than those in patient controls. These findings confirm that elevationd of GDNF^10^ and substance P^60^ are related to hypersensitivity in esophagus and might be caused by the increase of NHE3. Further study for verifying this will be required.

The limitations in this study are the small sample size and/or few sampling time points as well as the absence of protein data in esophageal tissues in all subjects. Because these clinical studies rely on the requests of subjects for examinations, an increase in the number of sampling time points would be difficult. The detection of protein would require enough biopsy tissues from the subjects, which could be stymied by the discomfort of subjects after multiple biopsies. Additionally, under the criticism of the institutional review board, it would be difficult to obtain more biopsy samples from each subject in a preclinical study.

In conclusion, this study found evidence for diurnal patterns of *TRPV1* and *NGF* mRNA expression in the human esophageal mucosa and tight interactions with the circadian-clock system. The findings of this study in relation to the well-established components of symptoms in GERD patients are summarized in a schematic diagram in Figure 3B: (1) acid injury or inflammation causes increased ATP and NGF release; (2) the circadian-clock system, including PER1, PER2, CRY2 and BMAL1, may be involved in this increased release through the regulation of protein expression in cell cycle or cell proliferation; (3) TRPV1 is activated and/or sensitized in the nerve fibers, and then the nerve fibers react to the increases in ATP and NGF release as well as GDNF uptake with increased persistent collateral sprouting; (4) the numbers of TRPV1 expressing nerve fibers increase, and all the nerve fibers exhibit a diurnal pattern of expression; and (5) central sensitization gradually increases and expresses as diurnal discomfort. These steps suggest that the circadian rhythm in the esophagus of GERD patients have responded to erosive damage and/or important to the mediation of therapeutic treatments.

## Additional Information

**How to cite this article**: Yang, S.-C. *et al.* Changes in Gene Expression Patterns of Circadian-Clock, Transient Receptor Potential Vanilloid-1 and Nerve Growth Factor in Inflamed Human Esophagus. *Sci. Rep.* **5**, 13602; doi: 10.1038/srep13602 (2015).

## Supplementary Material

Supplementary Information

## Figures and Tables

**Table 1:** Sequences of primers for real-time quantitative PCR.

Gene	Primer sequence	Accession number
β-actin	F, 5′CTCCTCCTGAGCGCAAGTACTC3′	NM_001101
	R, 5′CTGCTTGCTGATCCACATCTG3′	
PER1	F, 5′CCCAGCACCACTAAGCGTAAA3′	NM_002616
	R, 5′TGCTGACGGCGGATCTTT3′	
PER2	F, 5′GCTGGCCATCCACAAAAAGA3′	NM_022817
	R, 5′GCGAAACCGAATGGGAGAAT3′	
PER3	F, 5′GCCTTACAAGCTGGTTTGCAA3′	NM_016831
	R, 5′CTGTGTCTATGGACCGTCCATTT3′	
BMAL1	F, 5′GCCGAATGATTGCTGAGG3′	NM_001178
	R, 5′CACTGGAAGGAATGTCTGG3′	
CLOCK	F, 5′ACGACGAGAACTTGGCATTG3′	NM_004898
	R, 5′TCCGAGAAGAGGCAGAAGG3′	
CRY1	F, 5′ACTCCCGTCTGTTTGTGATTCG3′	NM_004075
	R, 5′GCTGCGTCTCGTTCCTTTCC3′	
CRY2	F, 5′TCTTCCAGCAGTTCTTCC3′	NM_021117
	R, 5′GTAGTCCACACCAATGATG3′	
TIM	F, 5′CAGCACCACCAGGACAAGC3′	NM_003920
	R, 5′GCAGATTGCCAAAACAGAGCA3′	
CB1	F, 5′CCAGAAGAGCATCATCATC3′	NM_033181
	R, 5′CCCAAAGACATCATACACC3′	
CB2	F, 5’GAAGATTGGCAGCGTGAC3′	NM_001841
	R, 5′TGTAGGAAGGTGGATAGCG3′	
NHE1	F, 5′TCAACAAGTTCCGTATCG3′	NM_003047
	R, 5′AAGATGACAGTGATGATGG3′	
NHE3	F, 5′GCACCACCATCATCGTAG3′	NM_004174
	R, 5′TCGCTCCTCTTCACCTTC3′	
TRPV1	F, 5′GAGTTTCAGGCAGACACTGGAA3′	NM_080704
	R,5′CTATCTCGAGCACTTGCCTCTCT3′	
NGF	F, 5′AGCAAGCGGTCATCATCC3′	NM_002506
	R, 5′GTGGCGGTGGTCTTATCC3′	
GDNF	F, 5′CTTGGGTCTGGGCTATGAAAC3′	NM_000514
	R, 5′CAAAGGCGATGGGTCTGC3′	
TAC1	F, 5′CTGAATTACTGGTCCGACTG3′	NM_013998
	R, 5′AGAACTGCTGAGGCTTGG3′	

F, forward; R, reverse; PER, period; TIM, Timeless; BMAL1, brain and muscle aryl-hydrocarbon receptor nuclear translocator-like protein-1; CLOCK, circadian locomotor output cycles kaput; CRY, cryptochrome; CB, cannabinoid receptors; NHE, Na/H exchangers; TRPV, transient receptor potential vanilloid; NGF, nerve growth factor; GDNF, glial derived neurotrophic factor; TAC1, protachykinin-1.

**Table 2:** Subject characteristics.

Characteristics	Patient controls	GERD patients	Difference
Patients, n	26	40	
Males/Females	12/14	24/16	*P* > 0.05
Age (range; mean)	18–46; 44.9	20–80; 46.9	*P* > 0.05
LA Grade			
None	26		
A		21	
B		10	
C		8	
D		1	
Biopsy time			
AM (9 AM)	17	25	
PM (4 PM)	9	15	

**Table 3:** Cluster correlations of gene expression within the group of genes that showed a rhythmic pattern in all subjects.

	*PER1*	*PER2*	*TRPV1*	*NGF*	*CRY2*
*PER1*		r = 0.1258	r = 0.5329	r = 0.6103	r = 0.1874
		*P* = 0.4454	*P* < 0.0001	*P* < 0.0001	*P* = 0.2532
		NS	**	***	NS
*PER2*	r = 0.1258		r = 0.4745	r = 0.0849	r = 0.4691
	*P* = 0.4454		*P* = 0.0002	*P* = 0.6074	*P* = 0.0026
	NS		***	NS	**
*TRPV1*	r = 0.5329	r = 0.4745		r = 0.5630	r = 0.5519
	*P* < 0.0001	*P* = 0.0002		*P* < 0.0001	*P* = 0.0003
	**	***		***	***
*NGF*	r = 0.6103	r = 0.0849	r = 0.5630		r = 0.5198
	*P* < 0.0001	*P* = 0.6074	*P* < 0.0001		*P* = 0.0007
	***	NS	***		***
*CRY2*	r = 0.1874	r = 0.4691	r = 0.5519	r = 0.5198	
	*P* = 0.2532	*P* = 0.0026	*P* = 0.0003	*P* = 0.0007	
	NS	**	***	***	

PER, period; TRPV, transient receptor potential vanilloid; NGF, nerve growth factor; CRY, cryptochrome.

**Table 4:** Cluster correlations of gene expressions within the group of genes without a rhythmic pattern in all subjects.

	*CRY1*	*TIM*	*CB1*	*GDNF*	*NHE3*	*TAC1*
*CRY1*		r = 0.4584	r = 0.6779	r = 0.3272	r = 0.3337	r = 0.3337
		*P* = 0.0043	*P* < 0.0001	*P* = 0.0481	*P* = 0.0436	*P* = 0.0436
		**	***	*	*	*
*TIM*	r = 0.4584		r = 0.5665	r = 0.5208	r = 0.4962	r = 0.3468
	*P* = 0.0043		*P* = 0.0003	*P* = 0.0010	*P* = 0.0018	*P* = 0.0355
	**		***	***	**	*
*CB1*	r = 0.6779	r = 0.5665		r = 0.3533	r = 0.4048	r = 0.2653
	*P* < 0.0001	*P* = 0.0003		*P* = 0.0319	*P* = 0.0130	*P* = 0.1126
	***	***		*	*	NS
*GDNF*	r = 0.3272	r = 0.5208	r = 0.3533		r = 0.7871	r = 0.4260
	*P* = 0.0481	*P* = 0.0010	*P* = 0.0319		*P* < 0.0001	*P* = 0.0086
	*	***	*		***	**
*NHE3*	r = 0.3337	r = 0.4962	r = 0.4048	r = 0.7871		r = 0.6165
	*P* = 0.0436	*P* = 0.0018	*P* = 0.0130	*P* < 0.0001		*P* < 0.0001
	*	**	*	***		***
*TAC1*	r = 0.3337	r = 0.3468	r = 0.2653	r = 0.4260	r = 0.6165	
	*P* = 0.0436	*P* = 0.0355	*P* = 0.1126	*P* = 0.0086	*P* < 0.0001	
	*	*	NS	**	***	

CRY, cryptochrome; TIM, Timeless; CB, cannabinoid receptors; GDNF, glial derived neurotrophic factor; NHE, Na/H exchangers; TAC1, protachykinin-1.
