# Supplementary material for: Changes in Gene Expression Patterns of Circadian-Clock, Transient Receptor Potential Vanilloid-1 and Nerve Growth Factor in Inflamed Human Esophagus
Source: Sci Rep. 2015 Sep 4;5:13602. doi: 10.1038/srep13602 (PMC4559770; doi:10.1038/srep13602)
Supplement: Supplementary Information [file srep13602-s1.pdf]

Changes in Gene Expression Patterns of Circadian-Clock, Transient Receptor Potential Vanilloid-1 and Nerve Growth Factor in Inflamed Human Esophagus

Shu-Chuan Yang, Chien-Lin Chen, Chih-Hsun Yi, Tso-Tsai Liu, Kun-Ruey Shieh*

Figure S1. Correlations among levels of *PER1* (period1), *PER2*, *CRY2* (cryptochrome 2), *TRPV1* (transient receptor potential vanilloid receptor 1) and *NGF* (nerve growth factor) mRNA expression in all subjects are shown.

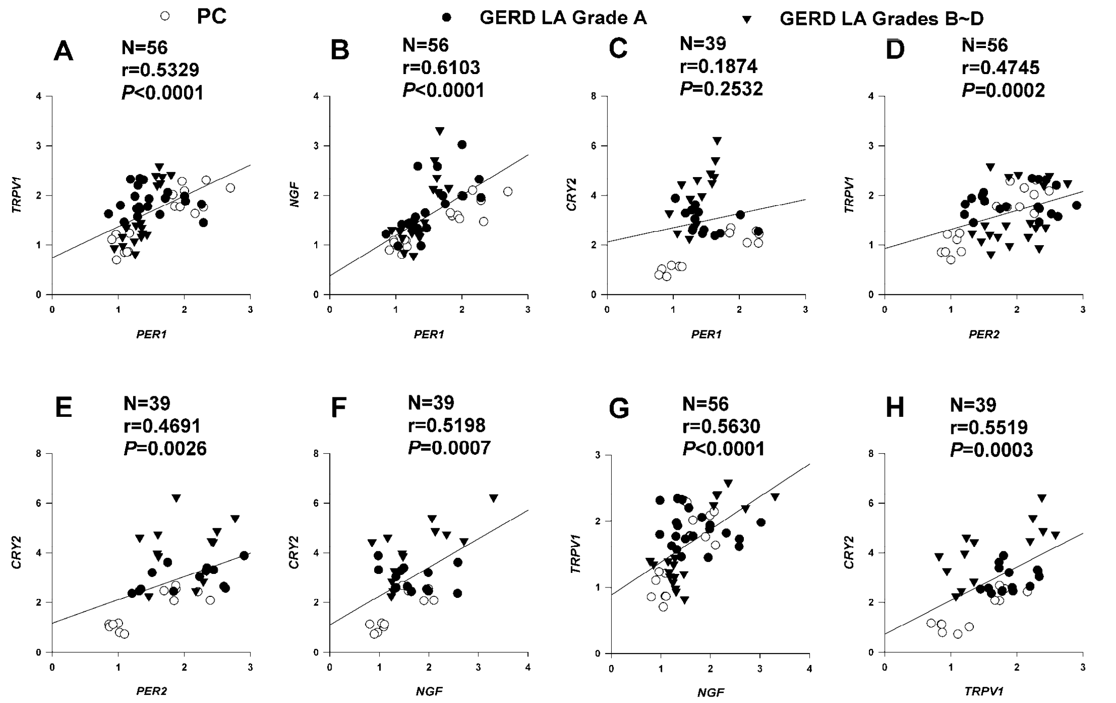

PER1 mRNA expression was correlated well with (A) *TRPV1* expression (r=0.5329, *P*<0.0001) and (B) *NGF* expression (r=0.6103, *P*<0.0001), but not with (C) *CRY2* expression (r=0.1874, *P*=0.2532). *PER2* mRNA expression was correlated well with (D) *TRPV1* expression (r=0.4745, *P*=0.0002) and (E) *CRY2* expression (r=0.4691, *P*=0.0026). *NGF* mRNA expression was correlated well with (F) *CRY2* expression (r=0.5198, *P*=0.0007) and (G) *TRPV1* expression (r=0.5630, *P*<0.0001). *TRPV1* mRNA expression was correlated well with (H) *CRY2* expression (r=0.5519, *P*=0.0003). Open circle, filled circle and inverted triangle indicated patient controls (PC), the patients with Los Angeles (LA) classification grade (A) gastroesophageal reflux disease (GERD LA Grade A), and the patients with LA classification grades (B-D) GERD (GERD LA Grades B-D), respectively. Each dot represents datum for an individual subject.

Figure S2. Correlations among levels of *CRY1* (cryptochrome 1), *TIM* (timeless), *CB1* (cannabinoid receptor 1), *GDNF* (glial derived neurotrophic factor), *NHE3* (Na/H exchanger 3), and *TAC1* (protachykinin-1) mRNA expression in all subjects were shown.

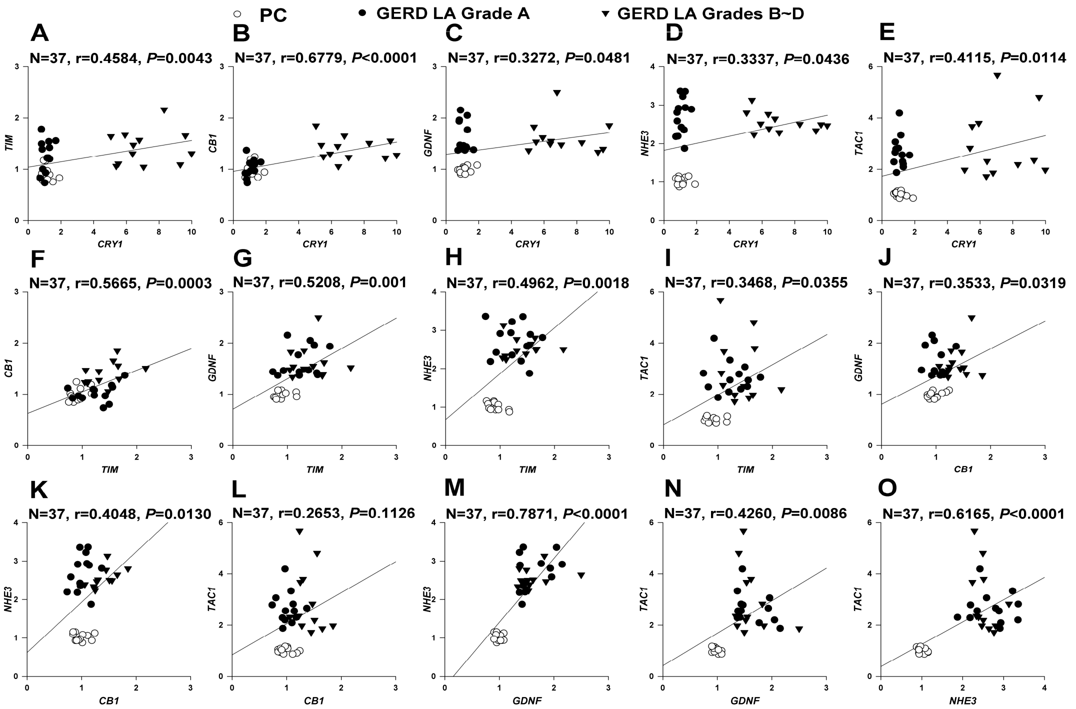

CRY1 mRNA expression was correlated well with (A) *TIM* expression (r=0.4584, *P*=0.0043), (B) *CB1* expression (r=0.6779, *P*<0.0001), (C) *GDNF* expression (r=0.3272, *P*=0.0481), (D) *NHE3* expression (r=0.3337, *P*=0.0436) and (E) *TAC1* expression (r=0.4115, *P*=0.0114). *TIM* mRNA expression was correlated well with (F) *CB1* expression (r=0.5665, *P*=0.0003), (G) *GDNF* expression (r=0.5208, *P*=0.001), (H) *NHE3* expression (r=0.4962, *P*=0.0018) and (I) *TAC1* expression (r=0.3468, *P*=0.0355). *CB1* mRNA expression was correlated well with (J) *GDNF* expression (r=0.3533, *P*=0.0319) and (K) *NHE3* expression (r=0.4048, *P*=0.013), but not with (L) *TAC1* expression (r=0.2653, *P*=0.1126). *GDNF* mRNA expression was correlated well with (M) *NHE3* expression (r=0.7871, *P*<0.0001) and (N) *TAC1* expression (r=0.4260, *P*=0.0086). *NHE3* mRNA expression was correlated well with (O) *TAC1* expression (r=0.6165, *P*<0.0001). Open circle, filled circle and inverted triangle indicated patient controls (PC), the patients with Los Angeles (LA) classification grade (A) gastroesophageal reflux disease (GERD LA Grade A), and the patients with LA classification grades (B-D) GERD (GERD LA Grades B-D), respectively. Each dot represents datum for an individual subject.
